# Comparative Study of Electrophoretic Deposition of Doped BaCeO_3_-Based Films on La_2_NiO_4+δ_ and La_1.7_Ba_0.3_NiO_4+δ_ Cathode Substrates

**DOI:** 10.3390/ma12162545

**Published:** 2019-08-09

**Authors:** Elena Kalinina, Elena Pikalova, Alexandr Kolchugin, Nadezhda Pikalova, Andrey Farlenkov

**Affiliations:** 1 Laboratory of Complex Electrophysic Investigations, Ural Branch, Russian Academy of Sciences, Institute of Electrophysics, Yekaterinburg 620016, Russia; 2 Department of Physical and Inorganic Chemistry, Institute of Natural Sciences and Mathematics, Ural Federal University, Yekaterinburg 620002, Russia; 3 Laboratory of Solid State Oxide Fuel Cells, Ural Branch, Russian Academy of Sciences, Institute of High Temperature Electrochemistry, Yekaterinburg 620137, Russia; 4 Department of Environmental Economics, Graduate School of Economics and Management, Ural Federal University, Yekaterinburg 620002, Russia; 5 Laboratory of Materials and Devices for Clean Power Industry, Institute of Chemical Engineering, Ural Federal University, Yekaterinburg 620002, Russia; 6 Department of Technical Physics, Institute of Physics and Technology, Ural Federal University, Yekaterinburg 620002, Russia

**Keywords:** SOFC, proton-conducting electrolyte, thin-film technology, cathode substrate, electrophoretic deposition, stable suspension, Ba loss, protective layer, electrical conductivity

## Abstract

This paper presents the results of a comparative study of methods to prevent the loss of barium during the formation of thin-film proton-conducting electrolyte BaCe_0.89_Gd_0.1_Cu_0.01_O_3−δ_ (BCGCuO) on La_2_NiO_4+δ_-based (LNO) cathode substrates by electrophoretic deposition (EPD). Three different methods of the BCGCuO film coating were considered: the formation of the BCGCuO electrolyte film without (1) and with a protective BaCeO_3_ (BCO) film (2) on the LNO electrode substrate and the formation of the BCGCuO electrolyte film on a modified La_1.7_Ba_0.3_NiO_4+δ_ (LBNO) cathode substrate (3). After the cyclic EPD in six stages, the resulting BCGCuO film (6 μm) (1) on the LNO substrate was completely dense, but the scanning electron microscope (SEM) analysis revealed the absence of barium in the film caused by its intensive diffusion into the substrate and evaporation during the sintering. The BCO layer prevented the barium loss in the BCGCuO film (2); however, the protective film possessed a porous island structure, which resulted in the deterioration of the film’s conductivity. The use of the modified LBNO cathode also effectively prevented the loss of barium in the BCGCuO film (3). A BCGCuO film whose conductivity behavior most closely resembled that of the compacts was obtained by using this method which has strong potential for practical applications in solid oxide fuel cell (SOFC) technology.

## 1. Introduction

Recent trends in the development of solid oxide fuel cells (SOFC) are aimed at reducing the operating temperature to 600 °C and lower. For the efficient functioning of SOFC under such conditions, it is necessary to select functional materials which have acceptable conductivity values in the considered temperature range, as well as chemical and mechanical stability and compatibility. In addition, it is necessary to develop inexpensive and flexible methods for the formation of SOFC structures, including thin-film technologies.

Materials based on barium cerate, BaCeO_3_ (BCO), are prospective proton-conducting solid electrolytes [1]. A characteristic feature of these solid electrolytes is that, by means of acceptor doping, high values for the total conductivity can be achieved with minimal electronic conductivity contribution and low values of the activation energy. It is therefore advantageous to use these materials in the intermediate and low temperature hydrogen-fueled SOFCs relative to the number of proton conductors studied to date, as well as to oxygen-ion conducting electrolytes due to more efficient fuel utilization, as shown in a number of reviews and research papers [2,3,4,5].

The development of thin-film technology using various methods (spray pyrolysis, pulsed laser deposition, dip and spin coating, atomic layer deposition, copressing and calendering) for the formation of films based on doped barium cerate has been the subject of several studies [6,7,8,9,10,11,12,13]. Electrophoretic deposition (EPD) is a colloidal method applied for the formation of thin film, which is the most technologically flexible. Namely, the method is instrumentally simple, practically indifferent to the shape of the surface to be coated, has high productivity, and is well adapted for mass production [14,15,16]. It was shown in some recent works that this method can be successfully applied to the formation of thin films based on both oxygen-ion [17,18,19,20,21,22] and proton-conducting electrolytes [23,24,25,26]. However, there are few studies on formation of BCO-based thin-film electrolytes using EPD on cathode substrates.

In a recent paper [27] from our scientific group, the main features of the stable suspension preparation and the EPD process of doped barium cerate films on a highly conducting La_2_NiO_4+δ_ (LNO) cathode substrate was considered. Using ultrasonic processing and centrifugation, stable suspensions based on Sm and Gd doped BCO with high values of zeta potential and a narrow particle size distribution were obtained. The EPD and sintering modes were established to obtain dense electrolyte films of approximately 5 μm in thickness at 1500 °C. It was shown, however, that in contrast to the compact samples, the spatial distribution of elements in the sintered films was quite different from the nominal composition and characterized by a lack of barium. The probable reason for this is the more intense evaporation of barium from the film compared to that of the compacts due to the more developed surface. In the literature, the problem of Ba evaporation is considered for both volume samples and films [28,29,30,31,32,33,34]. It becomes more pronounced at increased sintering temperatures and for films with a thickness of less than 10 microns. In order to reduce the loss of barium, along with increasing Ba content in the initial composition [33], it was proposed to use different Ba sources during sintering: Ba-containing electrolyte placed on top of the film surface [29] or the application of protective films [30]. In addition, there are problems with instability of BCO-based films in anode conditions and their interaction with Ni-cermet anodes, which can also be resolved by means of the deposition of thin protective layers [30,35,36]. Fabbri at al. applied Y-doped BaZrO_3_ (BZY) film by pulsed laser deposition to protect Y-doped BaCeO_3_ (BCY) electrolyte on the anode side: the conductivity of the bilayer film was slightly lower than that of the BCY electrolyte. The modified cell performance, however, significantly decreased and reached only 20 mV/cm^2^ at 700 °C [35]. Bi & Traversa [36] used protective films made of the stable BaZr_0.7_Pr_0.1_Y_0.2_O_3−δ_ composition by a copressing method, on both the anode and cathode sides: the conductivity of the sandwiched electrolyte was 4 × 10^−3^ S/cm at 600 °C and the cell performance reached 185 mV/cm^2^ at 700 °C. The use of the Zr-free protective film (BaCeO_2_) [30] resulted in the increased cell performance 416 mV/cm^2^ at 700 °C along with the enhanced long-term stability of the cell. EPD was shown to be promising for the deposition of multilayer structures with easily controlled thickness of layers and good adherence between them [37]. Cyclic deposition was also mentioned among the methods to obtain electrolyte films with compositions close to those of the nominal ones [22,34].

Another possible reason for decreasing the Ba content in the films is its intensive diffusion into the electrode substrate. It was reported that a Ba-doped LNO cathode demonstrated reduced interaction with the BCGCuO electrolyte and showed superior electrochemical activity over long-term testing [38]. Thus, its use as a substrate could also be possible.

The purpose of this study was to obtain a BCO-based thin-film electrolyte on a cathode substrate using various methods to prevent the loss of Ba during the film sintering and to bring its electrical characteristics as close as possible to those obtained for compact samples. For the deposition, the BaCe_0.89_Gd_0.1_Cu_0.01_O_3−δ_ (BCGCuO) electrolyte was chosen due to its excellent electrical and sintering properties revealed in different studies [8,27,39]. Three different methods of BCGCuO film formation by the EPD method were considered: the formation of the BCGCuO electrolyte film without and with a protective BCO film on the LNO electrode substrate and the formation of the BCGCuO electrolyte film on a modified La_1.7_Ba_0.3_NiO_4+δ_ (LBNO) cathode substrate. As a result of the experiments, it was established that it is the diffusion of barium into the substrate that is the decisive factor in decreasing the barium content in the film. The cationic composition and electrical properties of the BCGCuO film were obtained on the Ba-doped LNO—the values of conductivity and activation energy were shown to be close to those of the compact sample. The results obtained are important for the development of the EPD method for producing thin electrolyte films on the basis of doped barium cerate and its structural analogs in SOFC technology.

## 2. Materials and Methods

### 2.1. Synthesis of The Electrolyte and Electrode Powders and Their Characterization

The synthesis of BaCe_0.89_Gd_0.1_Cu_0.01_O_3−δ_ (BCGCuO) was carried out via a nitrate combustion method using as the initial components BaCO_3_ (98.4% purity, JSC “Vekton”, St. Petersburg, Russia), Ce(NO_3_)_3_·6H_2_O (99%, SPA “Reaktiv”, Novosibirsk, Russia), Gd(NO_3_)_3_∙6H_2_O (99%, SPA “Reaktiv”, Novosibirsk, Russia) and CuO (99%, LLC JSC “Reakhim”, Moscow, Russia) by adding nitric acid (pH = 2) under heating. As a result of the preliminary experiments, the optimal fuel and oxidizer (glycine and citric acid) content was chosen, respectively, to be equal to 0.7 and 0.8 in the molar ratio to the metal cations. After completion of the redox reaction, the product was crushed in an agate mortar and annealed at 700 °C (10 h) and 900 °C (10 h) with a heating/cooling rate of 5 °C/min (with intermediate grinding). After the last synthesis stage, the material was milled for 2 h in a Fritsch Pulverisette planetary mill with zirconia balls in a polytetrafluorethylene (PTFE) drum in an isopropyl alcohol medium.

The synthesis of BaCeO_3_ (BCO) was performed via a nitrate combustion method using Ba(NO_3_)_2_∙4H_2_O (98%, SPA “Reaktiv”, Novosibirsk, Russia) and Ce(NO_3_)_3_·6H_2_O (99%, SPA “Reaktiv”, Novosibirsk, Russia) as starting components. Citric acid was added in a molar ratio of 2:1 to the metal cations. Nitrates and citric acid were dissolved in distilled water at 80 °C. Following that, a 10 vol. % ammonia solution was added dropwise until the solution medium was slightly acidic (pH = 6). The resulting solution was heated up to the temperature of 280 °C, at which the processes of water evaporation, the formation of a gel-like residue, its ignition, and the formation of highly dispersed ash successively occurred. The prepared powders were ground in the agate mortar and annealed at 1050 °C (5 h) and at 1150 °C (5 h) with a heating/cooling rate of 5 °C/min. After the last stage of the synthesis, the material was milled for 2 h in the planetary mill.

A two-stage solid state reaction method [40] was used to obtain La_2_NiO_4+δ_ (LNO) and La_1.7_Ba_0.3_NiO_4+δ_ (LBNO) materials for the cathode substrates to decrease their sinterability and maximally reconcile the substrate and the electrolyte film sintering temperatures. The initial components, La_2_O_3_ (99.99%, JSC “Vekton”, St. Petersburg, Russia), NiO (98.4%, JSC “Vekton”, St. Petersburg, Russia) and BaCO_3_ (98.4%, JSC “Vekton”, St. Petersburg,, Russia), were mixed in the planetary mill (1 h) and calcined at 1150 °C (2 h) with a heating/cooling rate of 5 °C/min with following milling in a planetary mill (1 h). Final synthesis was carried out at 1250 °C, 5 h; after that, the materials were milled for 1 h in the planetary mill.

The specific surface area of the BCGCuO, BCO, LNO, and LBNO powders was determined by the method of low temperature nitrogen adsorption (Brunauer-Emmett-Teller (BET) method) using a TriStar 3000 (Micromeritics, Norcross, USA) device. X-ray phase analysis of the powders obtained was performed using a D/MAX-2200 (RIGAKU, Tokyo, Japan) diffractometer with a copper radiation source (CuKα) at a wavelength λ = 1.54056 Å in the angle range of 20° ≤ 2θ ≤ 80°. The identification of the phase composition and the crystal structure was carried out by means of the JCPDS (Joint Committee on Powder Diffraction Standards) base using MDIJade 6.5 (Livermore, CA, USA) software. The chemical composition of the BCGCuO powder was verified by inductively coupled plasma optical emission spectroscopy using an Optima 4300 DV (Perkin Elmer, Waltham, USA) device. The microstructure of the BCGCuO powder was examined using a JSM-6390 LA (JEOL, Tokyo, Japan) scanning electron microscope. The microstructure of the BCO powder was examined using a Mira 3 LMU (Tescan, Brno, Czech Republic) scanning electron microscope.

### 2.2. Suspension Preparation and Characterisation

Suspensions for the EPD were prepared on the basis of the milled BCO and BCGCuO powders in a mixed dispersion medium of isopropanol (special purity grade, JSC «Component-Reaktiv», Moscow, Russia) /acetylacetone (analytically pure grade, Merck, CAS 123-54-6) (70/30 vol. ratio). The suspensions with concentrations of 10 g/L were prepared by accurately weighing the powder, mixing with the dispersion medium, and ultrasonic treatment using an ultrasonic bath UZV-13/150-TH («Reltec», Yekaterinburg, Russia) for 5–125 min with control of the average particle and aggregate size. Removal of large aggregates intact during the ultrasonic treatment was performed by centrifuging using a Z383 (Hermle Labortechnik GmbH, Wehingen, Germany) centrifuge at a speed of 1500 rpm for 3 min. Then, the suspension was carefully removed with a syringe from the sediment. The concentration of the disaggregated suspensions was 7 g/L. Particle size distribution and zeta potential values in the suspensions of micro-sized BCO and BCGCuO powders were measured by dynamic and electrophoretic light scattering methods using a ZetaPlus (Brookhaven Instruments Corporation, NY, USA) particle analyzer. All measurements were carried out in isothermal conditions in air at 25 °C.

### 2.3. Electrophoretic Deposition

Electrophoretic deposition was performed on a specialized computerized installation, providing constant current or constant voltage modes, which was developed in the Laboratory of Pulse Process, Institute of Electrophysics, UB RAS. For the formation of the electrode substrates, the powders were pressed into disks by the method of semidry pressing with the addition of polyvinyl butyral binder at a pressure of 5 t/cm^2^. The sintering of the substrates was carried out at a temperature of 1450 °C, 3 h, with a heating/cooling rate of 5 °C/min. The substrates were polished, cleaned in the ultrasonic bath, and annealed at 900 °C for 1 h before their usage for the EPD. The relative density of the substrates, determined from their dimensions and weight, was ~80% of the theoretical density calculated according to X-ray diffraction data. The LNO and LBNO substrates with an effective area of 12 mm^2^ were used as a cathode for the EPD. A stainless steel disk of the same size was used as an anode, the distance between the electrodes was 1 cm. Electrophoretic deposition of the electrolyte films on the cathode substrates was carried out by deposition/sintering cycles from the stable suspensions obtained at a constant voltage of 80 V and a deposition current in the range from 1.00 to 1.21 mA/cm^2^. Butyl methacrylate copolymer containing 5 mol. % of methacrylic acid (BMC-5, JSC “NPK Izomer”, Dzerzhinsk, Russia) was added to the suspension to avoid cracking of the deposited coatings during drying [21]. The deposition time in each cycle was 1–3 min. The weight of the green layer in each cycle was 0.4–0.7 mg/cm^2^ of the geometric surface of the cathode. After the deposition, the electrolyte layer was compacted in a centrifuge at a speed of 1000 rpm for 2 min and dried at 25 °C in a Petri dish to slowly remove the solvent and then sintered. To prevent the film swelling due to the fast evacuation of the gaseous products of the organic components’ burning, a prolonged sintering mode was used, during which a residual solvent was removed up to about 250 °C and then a binder was burned out in the range of 250–400 °C. The sintering of the film structures was carried out at temperatures of 900–1350 °C, 2 h, at the intermediate stages of cyclic deposition and for 1450 °C, 2 h, at the final stage. The rate of heating and cooling was 1 °C/min.

The microstructure of the deposited film was studied by means of the scanning electron microscope Mira 3 LMU (Tescan, Brno, Czech Republic) equipped with the Oxford Instruments INCA Energy 350 X-Ray energy-dispersive (EDX) microanalysis system with an X-max 80 detector (Oxford Instruments, Abingdon, UK) and Aztec 3.1 (Oxford Instruments, Abingdon, UK) software for EDX-data treatment. SEM images in BSE (back-scattered electrons) mode were obtained at a high voltage of 10 kV and beam intensity of 10. The high voltage of 20 kV and the beam intensity of 15 were used for the EDX analysis. The sputtering of the conducting carbon coatings (<10 nm) was performed using the Q150T ES (Quorum Technologies, Lewes, UK) system. To prepare the cross-section of the cells for the film microstructure characterization, a MetPrep 4/PH-4 (Allied, Compton, CA, USA) polishing machine with diamond suspensions was used.

### 2.4. Conductivity Measurements

Compact bar-shaped samples were prepared from the BCGCuO, LNO, and LBNO powders by the method of semidry pressing with the addition of polyvinyl butyral binder at a pressure of 5 t/cm^2^ with sintering at 1450 °C for 5 h. Relative densities of the samples were in the range of 92%–94%. The measurement of the conductivity of the compact samples was carried out by a four-probe method under a direct current in air in the temperature range of 400–850 °C. Current and potential probes on the samples were made from 0.2 mm diameter platinum wire. In order to improve the contact, fine platinum paste was painted along the contact of the probes with the sample and then sintered at 900 °C, 1 h.

To evaluate the conductivity of the BCGCuO electrolyte film deposited on the LBNO cathode substrate, the electrochemical cell Pt|LBNO|BCGCuO|Pt was fabricated. Platinum electrodes with an effective surface area of approximately 0.3 cm^2^ were deposited by painting and sintered at 950 °C, 1 h. After the first measurement Pr(NO_3_)_3_ was used for the Pt electrode activation. The porous Pt electrodes were impregnated with a saturated alcoholic solution of Pr(NO_3_)_3_·6H_2_O (99.9%, LLC “Krein”, Yekaterinburg, Russia) (210 g of praseodymium nitrate per 100 mL of ethanol). Decomposition of the nitrate to the oxide was performed by calcination of the electrodes at 600 °C, 1 h, with a heating/cooling rate of 1.5 °C/min.

Electrochemical measurements on the obtained cell were performed by an impedance spectroscopy method using a potentiostat SI 1260 and an electrochemical interface SI 1287 (Solartron Analytical, Farnborough, Hampshire, UK) in the frequency range of 0.01 Hz–1 MHz with an amplitude of applied voltage of 30 mV and resolution of 30 points per decade. The sample was oppressed between two Pt grids with a thickness of 0.01 cm and a mesh size of 0.1 cm × 0.1 cm, which were connected to the measuring equipment by means of Pt wires via a two-electrode, four-cable mode which excludes the impedance of current-supplying cables and wires from the overall impedance of the system. Studies were conducted in the temperature range of 400–850 °C. The resistance of the bilayer system (the LBNO substrate with the BCGCuO electrolyte film) was determined from the analysis of the impedance spectra using the software Zview v. 2.8. From the calculated resistance the LBNO resistance measured by the four-probe method was subtracted, taking into account the geometric dimensions of the electrode substrate.

## 3. Results and Discussion

### 3.1. Crystal Structure Parameters and Microstructure of The Powders

According to the X-ray diffraction (XRD) data, after the final synthesis stage, the BCGCuO powder was single-phase (Figure S1, Supplementary materials) and possessed an orthorhombic structure (space group Pmcn), with lattice parameters a = 8.793(2) Å, b = 6.233(1) Å, c = 6.220(1) Å. Chemical composition of the BCGCuO powder was close to the nominal one (Table S1). The specific surface area of the BCGCuO powder after the final milling was approximately 3 m^2^/g and it was characterized by the presence of large loose agglomerates with a size of 1–10 μm and their clusters with a size of up to 30 μm. The BCO powder was single-phase (Figure S1, Supplementary materials) and had an orthorhombic structure (space group Pnma) with lattice parameters a = 8.807(1) Å, b = 6.214(4) Å, c = 6.219(7) Å. The specific surface area of the milled BCO powder also reached 3 m^2^/g. Both particle agglomerates with a size of 1–10 μm and big clusters with a size up to 50 μm were present in the BCO powder. The LNO and LBNO powders after the final synthesis at 1250 °C, according to the XRD study, were single phase (Figure S2) and had an orthorhombic structure (space group Fmmm) with lattice parameters a = 5.448 (1) Å, b = 5.477(1) Å, c = 12.667(8) Å and a tetragonal structure (space group I4/mmm) with lattice parameters a = 3.8494(7) Å, c = 12.8158(3) Å, respectively. Specific surface area of the electrode powders after the final synthesis stage and ball-milling was approximately 1 m^2^/g. Examples of the BCGCuO, BCO, and LNO powders’ microstructure are shown in Figure S3–S5. The average size of the particles calculated from the SEM images was 2 ± 1 µm, 20 ± 5 µm, and 4 ± 1 µm, for BCGCuO, BCO, and LNO, respectively.

### 3.2. Preparation of Stable Suspensions for The EPD

In order to successfully carry out the EPD process, it is necessary to ensure the stability of the suspensions used, which is usually characterized by the value of the zeta potential [14,41]. The stability of suspensions is largely influenced by the choice of dispersion medium. For the preparation of suspensions based on solid electrolytes, alcohols and ketones are mainly used, as well as mixtures thereof, to solvate the particle surface well [19,42,43,44,45]. Thus, the choice of the mixed isopropanol/acetylacetone dispersion medium in this study was made on the basis of the preliminary studies conducted in [27,45].

Within the suspension, competing processes of aggregation and disaggregation of particles occur; therefore, both aggregates and individual particles are present in the liquid medium. As a result, a certain size distribution of particles and their aggregates is determined in the suspension, characterized by the value of the average hydrodynamic particle diameter and the distribution width parameter (geometric standard deviation (GSD), a dimensionless value). The fractional composition of the suspension is determined by the bimodal distribution and the values of the average particle diameter of the individual fractions. These indicators of the suspension determine not only the kinetics of the EPD process, but also the morphology of the film deposited.

To ensure the uniformity of the coating, it is advisable to conduct the EPD from a suspension with the smallest possible width of size distribution. For this purpose, methods of disaggregation are used, such as ultrasonic treatment (UST) and methods of mechanical dispersing (long ball-milling [46,47], mixing in a magnetic stirrer [48] or processing in a dissolver [49]). In this study, a combination of UST and centrifugation was used, which, despite the short processing time, can significantly increase the proportion of individual particles and obtain narrow particle size distributions in the suspension. This will significantly improve the quality of the films produced [20,45].

**Figure 1:**
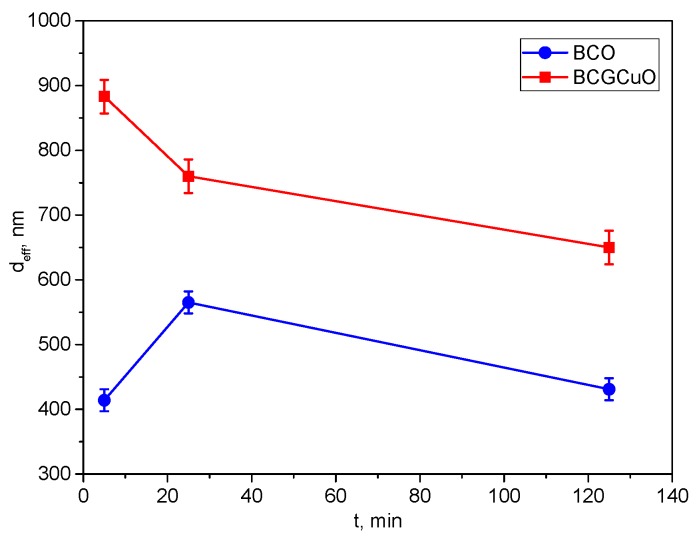
Dependence of the effective hydrodynamic size of the aggregates (d_eff_) on the time of the ultrasonic treatment of the suspensions of BaCe_0.89_Gd_0.1_Cu_0.01_O_3−δ_ (BCGCuO) and BaCeO_3_ (BCO) powders.

The results of the analysis of the dependence of the effective hydrodynamic diameter (d_eff_) of aggregates for the BCO suspension on the UST duration (Figure 1) showed that during the UST process the size of the aggregates in suspensions first increased sharply from 414(12) nm (5 min UST) to 565(17) nm (25 min UST), then gradually decreased to 431(13) nm after 125 min of UST. Thus, sonication does not have a remarkable influence on deaggregation of the BCO suspension. This behavior of the effective hydrodynamic diameter of the aggregates in the suspension is possibly related to the morphology of the initial BCO micro-sized powder. Contrarily, during the UST of the BCGCuO suspension, the size of the aggregates gradually decreased from 883(26) nm (5 min UST) to 650(20) nm (125 min UST). As a result of sonication, complete deaggregation of the BCGCuO suspension did not occur; however, the hydrodynamic size of the aggregates significantly decreased. Unbroken large aggregates present in both suspensions after UST can be separated by centrifugation. 

**Figure 2:**
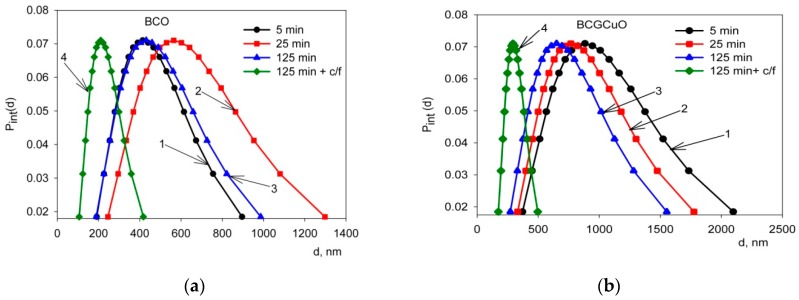
Unimodal distributions: (**a**) for the BCO suspension, UST 5 min (1), UST 25 min(2), UST 125 min (3), and ultrasonic treatment (UST) 125 min + centrifugation for 3 min at a speed of 1500 rpm (4); (**b**) for BCGCuO suspension, UST 5 min (1), UST 25 min(2), UST 125 min (3), and UST 125 min + centrifugation for 3 min at a speed of 1500 rpm (4), where Pint(d) is the probability density of the particle size distribution, d is the characteristic diameter of particles.

Figure 2 shows the unimodal distribution of the particles’ size according to the intensity scattering measurements of the BCO and BCGCuO suspensions after sonication for 5–125 min and after subsequent centrifugation for 3 min at a speed of 1500 rpm. It is seen that as the UST duration increases, the particle size distribution in the BCO suspension broadens. This is confirmed by the GSD value which is a parameter of the distribution width and associated with the standard deviation according to the formula σ = ln(GSD).

For the BCO suspension ultrasonically treated for 5 min, 25 min, and 125 min, the GSD was 1.60(7), 1.66(7), and 1.65(7), respectively. As noted earlier, the long-term UST did not affect the effective hydrodynamic diameter of the particles in the BCO suspension, which even increased slightly from 414(12) nm to 431(13) nm. After centrifugation, the particle size distribution became narrower with the effective hydrodynamic diameter of BCO particles and the GSD being 211(6) nm and 1.52(7)**,** respectively. This is associated with the removal of large particles during the centrifugation process.

For the BCGCuO suspension, the width of the distribution did not change with time of UST, which is confirmed by the GSD values. Thus, for the BCGCuO suspension treated with ultrasound for 5 min, 25 min, and 125 min, the GSD was 1.69(7), 1.66(7), and 1.69(7), respectively. The duration of the UST affected the effective hydrodynamic particle diameter of BCGCuO, which decreased from 883(26) nm to 650(20) nm. After centrifugation, the particle size distribution became narrower than for suspensions treated with ultrasound only. The effective hydrodynamic diameter of the BCGCuO and GSD particles was 294(9) nm and 1.37(6), respectively, which is associated with the removal of a fraction of large particles during the centrifugation process. The results of determining the fractional composition of bimodal distributions for the suspensions of BCO and BCGCuO powders are given in Table 1. 

It is seen from the results obtained that for the BCO suspension at 125 min UST, first, large aggregates of approximately 1116(33) nm were removed, which had appeared after 25 min of UST and whose share in the suspension was 94%. Secondly, only aggregates with a size of approximately 773(23) nm (96%) remained; the share of small aggregates with a size of 197(6) nm was 4%. After centrifugation, only aggregates with a size of 753(23) nm (88%) remained and particles with a size of 41(1) nm (12%) appeared. For the BCGCuO suspension after 25 min of UST, first, large aggregates of approximately 2473(74) nm in size, whose share in the suspension was 24%, were removed. Secondly, only the aggregates with a size of approximately 1384(42) nm (55%) remained, while the proportion of small aggregates with a size of 449(13) nm was 45%. UST during 125 min reduced the size of small aggregates to 370(11) nm (19%) but, at the same time, the size of large aggregates (1639(49) nm) and their share in the suspension (81%) increased. After centrifugation, only small aggregates 323(10) nm in size (81%) remained and particles of 60(2) nm in size (19%) appeared. Thus, the combination of UST and centrifugation allowed us to obtain suspensions of BCO and BCGCuO with narrow particle size distributions.

Zeta potential determines the value of the electrostatic repulsion between the particles in colloid systems. At sufficiently high values of the zeta potential, the electrostatic repulsion forces exceed the attractive Van der Waals forces; thus, the stability of the disperse phase in the colloidal system is ensured. When the zeta potential value is low, the attraction exceeds the repulsion and the stability of the suspension will be violated. Thus, colloids with high zeta potential are electrically stabilized, while colloids with low zeta potential tend to coagulate or flocculate. The value of the zeta potential being equal to 26 mV (positive or negative) can be considered as a characteristic value, determining the suspensions’ stability. The greater the zeta potential exceeds this value, the more stable the suspension [14,41,50].

Zeta potential measurements in the disaggregated suspensions of micro-sized BCO and BCGCuO powders were measured by the method of electrophoretic light scattering [41]. According to the data obtained, the BCO and BCGCuO suspensions had high positive zeta-potential values equal to +48(1) and +28(1) mV, respectively, which exceeded the characteristic value. Thus, the obtained results on the determination of the dispersity of the particles in the deaggregated suspensions of the micro-sized BCO and BCGCuO powders and measurements of their electrokinetic (zeta) potential showed the applicability of the suspension obtained to the implementation of the EPD process.

### 3.3. Electrophoretic Deposition of The Electrolyte Films and Their Characterization 

**Figure 3:**
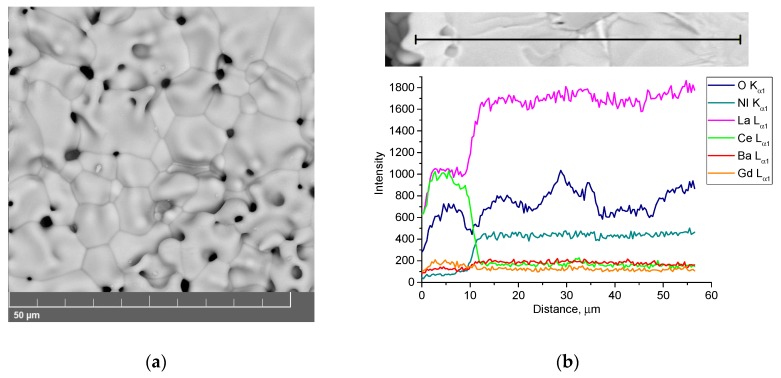

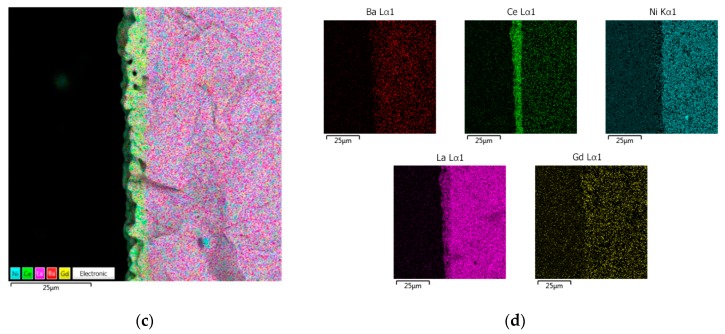
BCGCuO film structure on the LNO cathode (1450 °C, 2 h): (**a**) film surface; (**b**) distribution of the elements in the film/substrate system in dependence on the distance from the film surface and EDX mapping image: (**c**) integrated; (**d**) individual elements.

A BCGCuO electrolyte film with a relative weight of 3.7 mg/cm^2^ on an LNO substrate was obtained as a result of six cycles (First experiment (1)). The calculated thickness of the sintered film (provided that the BCGCuO theoretical density is 6.35 g/cm^3^) was 5.8 μm. In order to reduce the loss of barium in the BCGCuO film, sintering of the sample was carried out in a limited volume (closed crucible) with filling onto an electrophoretically deposited film of the BCGCuO powder. The profile of the film structure of BCGCuO/LNO sintered at 1450 °C, 2 h is shown in Figure 3. The film was dense with the presence of some pin-holes on the surface and its thickness was close to that which was calculated. 

The results of the EDX analysis of the film surface are shown in Figure S6 and Table S2. According to the EDX analysis data, Ba content in the BCGCuO film was close to zero. The presence of Ni in the film was not detected. Diffusion of Ba from the film into the substrate (Ba content in the substrate near the electrolyte/electrode interface ~0.8 at. %) (Figure S7, Table S3) and significant diffusion of lanthanum from the substrate into the film (~18 at. %) was observed. Thus, the usage of BCGCuO as a source of Ba in the first experiment was not effective and did not prevent the loss of barium. It is interesting to note that despite of decreasing the sintering temperature by 50 °C in comparison to that in [27], the BCGCuO film was dense with small surface defects (pin holes). It is probably that the shrinkage of the substrate, which was of 80% of relative density, facilitated the film densification. Further experiments with the substrate structure could help lower the sintering temperature and, therefore, prevent electrolyte-substrate interaction. This method was applied to decrease the interaction of EPD-deposited YSZ films with LaMnO_3_-based substrates [51,52,53].

**Figure 4:**
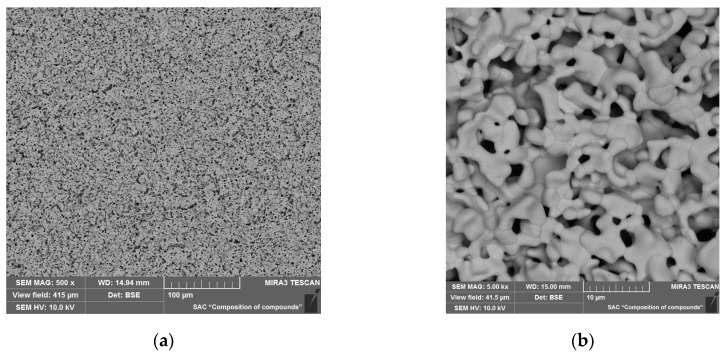

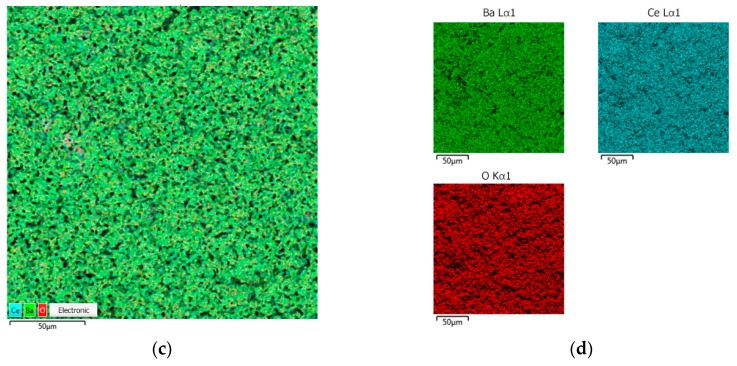
Scanning electron microscope (SEM) images of the surface of the two-layer BCGCuO/BCO film after the final sintering at 1450 °C, 2 h at different magnifications: (**a**) 500×; (**b**) 5000× and X-Ray energy-dispersive (EDX) mapping image: (**c**) integrated; (**d**) individual elements.

Based on the results obtained, we switched to another method for preserving barium in the BCGCuO film, namely, the application of a BCO protective film over the BCGCuO electrolyte film (the second experiment (2)). The two-layer BCGCuO/BCO film was formed according to the following scheme: first, BCGCuO layers were obtained by cyclic EPD according to the mode described above (five cycles with intermediate sintering at 900–1350 °C and final sintering at 1400 °C, 2 h) and then BCO layers were deposited onto the BCGCuO film (five cycles with intermediate sintering at 900–1350 °C). The whole structure was finally sintered at 1450 °C, 2 h. The total relative weight and calculated thickness of the two-layer BCGCuO/BCO film were 10.2 mg/cm^2^ and ~11 μm, respectively. In Figure 4a,b, the scanning electron images of the surface of the thin-film solid electrolyte BCGCuO with a protective film of BCO deposited on the cathode of LNO and sintered at a temperature of 1450 °C, 2 h are presented.

The surface of the BCGCuO/BCO film has a grainy structure with pores up to 10 microns in size (Figure 4b). As far as the deposition and sintering conditions for the BCGCuO film (first layer in the second experiment) were similar to those in the first experiment, such a grainy structure is possibly related to the morphology of the original micro-sized BCO powder. This is characterized by the presence of rather large sintered agglomerates which cannot be completely destroyed and removed by UST and centrifugation. According to the data on the spatial distribution of elements in the BCGCuO sample with a protective BCO film, the presence of only barium, cerium, and oxygen in the film was established (Figure 4c,d, Figure S8, Table S4). Due to the low surface conductivity of the BCO film, it was not possible to estimate the percentage of elements. Our results are quite different from those obtained in [30], where BCO-BaCe_0.8_Sm_0.2_O_3−δ_ (BCSO) film, deposited onto the NiO-BCSO cermet was sintered to a gas-tight state at 1400 °C. The authors reported that during sintering the diffusion of Ni from the anode substrate and Sm from the BCSO top-layer probably occurred, which could facilitate both the sintering of the BCO layer and increase its conductivity. In our case, the diffusion of Gd from the bottom electrolyte layer was not observed; therefore, the conductivity of undoped BCO would probably remain low and would block the electrolyte layer. 

At the next stage of the study, in order to to reduce the loss of barium in the film, the BCGCuO film was deposited on the modified LBNO cathode substrate (Third experiment (3)). The cyclic EPD of the film was performed according to the procedure described above. The relative weight of the green layer in each cycle was 0.6–1.8 mg/cm^2^ of the geometric surface of the cathode substrate. The number of cycles was increased to obtain a film of a relevant thickness. The total relative weight and estimated thickness of the BCGCuO film sintered at a temperature of 1450 °C, 2 h after three and eight deposition-sintering cycles was 1.2 mg/cm^2^ (1.9 μm) and 3.1 mg/cm^2^ (4.9 μm), respectively. 

According to the scanning electron microscopy data, the BCGCuO film after three cycles had an “island” structure: some areas with densely sintered grains and areas where the film did not completely cover the LBNO substrate were observed. It should be noted that after three cycles of EPD, Ba was detected in the film but there was also diffusion of La from the substrate into the electrolyte film. When the number of EPD cycles was increased up to eight, the porous structure of the film surface with some dense areas remained (grain and pore size up to 7 μm). The absence of Ni in the electrolyte film can be considered as a positive effect after the cyclic EPD. 

**Figure 5:**
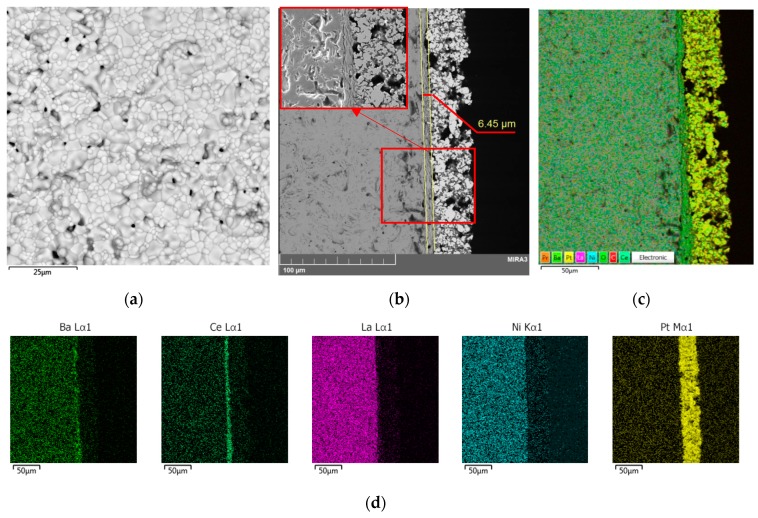
SEM images of the BCGCuO film deposited onto the La_1.7_Ba_0.3_NiO_4+δ_ LBNO substrate after the final sintering at 1450 °C, 2 h: (**a**) the film surface; (**b**) the cross-section of the BCGCuO film on the LBNO (on the left) substrate and Pt electrode (on the right); (**c**) integrated EDX mapping image of the cross section; (**d**) individual EDX mapping images of the cross section.

Further increasing the number of EPD cycles to twelve led to a decrease in the number of open pores and an increase in areas with a dense grain structure (Figure 5a). The total mass of the BCGCuO film after final sintering was 4.2 mg/cm^2^. It can be seen in the cross-section of the BCGCuO film with Pt electrode (Figure 5b,c) that the electrolyte film has uniform thickness and there are no defects in its volume. The calculated thickness of the sintered film is 6.6 μm, which is in good agreement with the results of the electron microscopy study (Figure 5b).

**Figure 6:**
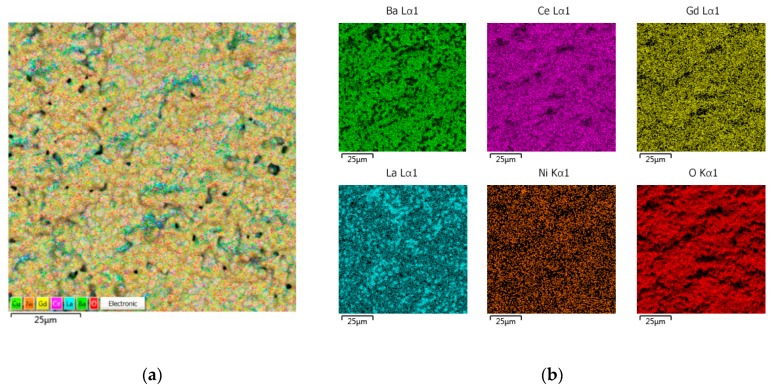
EDX mapping image the BCGCuO film deposited onto the LBNO substrate after the final sintering at 1450 °C, 2 h: (**a**) total; (**b**) individual elements.

The spatial distribution of elements in the BCGCuO film, presented in Figure 6, indicates the presence of Ba in the film. According to the EDX data, Ba content is very close to the nominal level (~17%). Partial diffusion of La into the film is evident and shows nonuniform behavior; however, the averaged La content in the film (~1%) is much lower than that in the case of the LNO substrate (~20%). No diffusion of Ni into the film was detected. It is interesting to note that the Gd content had also decreased. As far as this element is not prone to evaporation at increased temperature in contrast to Ba and Cu, the reason for such a decrease could be diffusion of Gd into the substrate. The average chemical composition of the film surface is presented in Table 2, the EDX spactra are shown in Figure S9. 

Nevertheless, the last method seems to be useful and Ba-doped substrates are effective for preventing Ba loss in the films of the base of BaCeO_3_ and its derivatives. In this way, conditions were established for the formation of the dense proton-conducting film with a thickness of less than 10 μm and a cationic composition close to the nominal composition of the initial powder on the cathode substrate. In the next chapter, the electrical properties of the film obtained were tested.

### 3.4. Electrical Properties of The Electrolyte BCGCuO Film Obtained by Cyclic EPD on the Cathode Substrates

**Figure 7:**
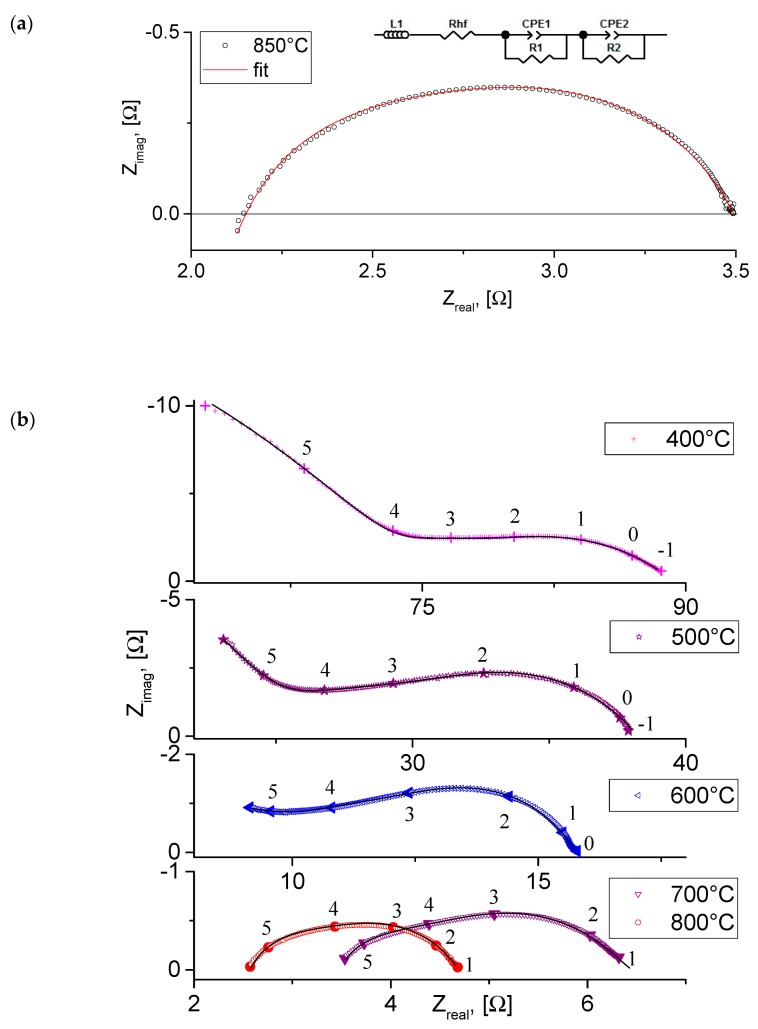
Typical impedance spectra of the Pt|LBNO|BCGCuO|Pt cell: (**a**) obtained at 850 °C in air; (**b**) the spectra view at different temperatures.

Figure 7 shows examples of the impedance spectra of the Pt|LBNO|BCGCuO|Pt cell with praseodymium nitrate-activated Pt electrodes obtained at a temperature of 850 °C (a) and the impedance diagrams obtained at different temperatures in air (b). The spectra present deformed semicircles and can be processed by an equivalent circuit consisting of the electrolyte resistance and several constant phase elements corresponding to the electrode process. At high temperatures of 850–700 °C, the high-frequency response in the spectra with characteristic capacitance value ~10^−6^ F/cm^2^ corresponds to the electrode process, namely charge transfer through the electrode-electrolyte interface. The electrolyte resistance corresponds to the intercept point of the high-frequency part of the spectrum with the real axis. Starting from the temperature of 650 °C, a high-frequency response in the spectra with characteristic capacitance C ~10^−9^ F/cm^2^ refers to the grain boundaries of the electrolyte. The total resistance of the electrolyte was determined from the fitting.

**Figure 8:**
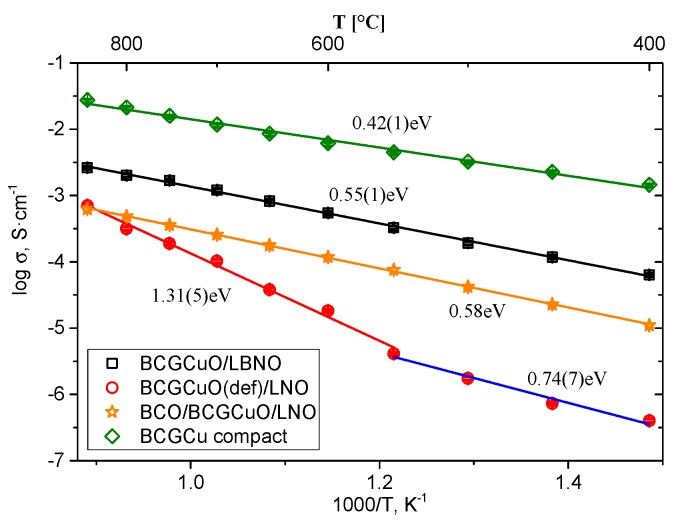
Arrhenius dependences of conductivity of the BCGCuO film deposited on the LBNO substrate, the deficient BCGCuO film deposited on the LNO substrate, the BCGCuO film with the protective BCO layer deposited on the LNO substrate and total conductivity of the BCGCuO compact sample.

Figure 8 shows the temperature dependences of the electrical conductivity of the BCGCuO film on the LBNO cathode and, for comparison, the electrical conductivity of the BCGCuO film with the BCO protective layer and the deficient BCGCuO film, measured using the same method. The total conductivity in air of the BCGCuO ceramic sample, measured by the four-probe method, is also presented.

Ba-deficient BCGCuO film shows electrical behavior quite different from that of the compact sample with very high values of the activation energy both in the high-temperature (1.31 eV) and the low-temperature (0.74 eV) range. The BCGCuO film with the BCO protective layer deposited on the LNO substrate and the BCGCuO film deposited on the LBNO susbtrate show similar behavior. The values of the apparent activation energy of the conductivity of these films are close to the value of the activation energy of conductivity for the compact BCGCuO sample (0.58 eV, 0.55 eV and 0.42 eV, respectively). The proximity of the activation energies of the electrolyte films and the electrolyte volume sample means that the electrolyte resistance values were extracted from the spectra correctly and the contribution of the contact resistance was negligible. 

Despite the chemical composition being close to that of the nominal one, the electrical conductivity of the film on the LBNO substrate at 600 °C in air (5.5 × 10^−4^ S/cm) was lower than that of the compact sample (6.9 × 10^−3^ S/cm). A probable reason for the decrease in the total conductivity value is the slight Ba deficiency of the film which usually has a significant impact on the electrical properties [33]. Diffusion of La into the film with partial susbstitution both in Ba and Ce position can also lower the conductivity compared to the Gd-doped BCO [54,55]. Nevertheless, since the modification of the substrate is a promising direction for the EPD deposition of the films based on doped barium cerate, our further research will be aimed at finding materials for the functional layer of substrates to further reduce the interaction with the film. For example, in recent studies, it was shown that such materials can be layered praseodymium or neodymium nickelates codoped with barium [56,57]. In addition, it is of interest to modify the film composition itself by introducing a small amount of zirconium which would increase the stability of the film in atmospheres containing hydrocarbons and water vapor [58,59] and to introduce an excess of Ba in the initial composition [33] or partlly substitute Ba with other alkaline earth metals to reduce its evaporation [32]. 

The BCGCuO film obtained in the second experiment (deposited on LNO substrate with BCO protective layer) has even low conductivity (1.2 × 10^−4^ S/cm at 600 °C). Probable reasons for that can be both low conductivity of the BCO film due to its morphology and features of the electrical behavior and La-diffusion into the electrolyte film from the LNO substrate, which is even higher than that in the case of the deposition on the LBNO substrate. This fact suggests that the use of protective layers could be more effective in combination with the structural modification of the substrate. The use of the nano-sized BCO for the deposition of the protective layer, for example, obtained by the laser evaporation method [21,22], may improve the structure and lower the thickness of the protective layer and, thus, improve the conductivity of the BCGCuO/BCO film. All these above-mentioned directions will be the subject of future studies.

## 4. Conclusions

In the present study, proton-conducting solid electrolyte BaCe_0.89_Gd_0.1_Cu_0.01_O_3−δ_ (BCGCuO) and BaCeO_3_ (BCO) were synthesized by the citrate-nitrate method. Aggregatively stable suspensions based on BCO and BCGCuO powders were prepared in a mixed dispersion medium of isopropanol-acetylacetone (70/30 vol. %) and showed high positive zeta potential values of +48(1) and +28(1) mV, respectively. It was shown that ultrasonic processing in combination with subsequent centrifugation yields a substantially narrow distribution of particle sizes. The characteristic features of micro-sized powders are the presence of irregular particles and sintered agglomerates. Consequantly, deposition/sintering cycles are required to obtain dense coatings on their base by the EPD method. It was shown that the most effective method to prevent the loss of barium in the BCGCuO film was deposition of the BCGCuO film on the modified LBNO cathode substrate. The modification of the substrate is a promising direction for the development of EPD films based on doped barium cerate and can be used in combination with deposition of protective layers. Our future research will be aimed at finding materials for the functional layer of substrates to further reduce the interaction with the film and enhance the film’s electrical properties.

## Figures and Tables

**Table 1:** The results of the fractional composition of suspensions of micro-sized BCO and BCGCuO powders in a mixed dispersion medium isopropanol/acetylacetone (70/30 vol. %).

Particles	Processing Method	First Fraction		Second Fraction	
		Average Size, nm	Content %	Average size, nm	Content %
BCO	UST 5 min	152(5)	13	580(17)	87
	UST 25 min	278(8)	6	1116(33)	94
	UST 125 min	197(6)	4	773(23)	96
	UST 125 min + Centrifugation for 3 min at a speed of 1500 rpm	41(1)	12	753(23)	88
BCGCuO	UST 5 min	465(14)	76	2473(74)	24
	UST 25 min	449(13)	45	1384(42)	55
	UST 125 min	370(11)	19	1639(49)	81
	UST 125 min + Centrifugation for 3 min at a speed of 1500 rpm	60(2)	19	323(10)	81

**Table 2:** The averaged elemental composition of the BCGCuO film on the modified cathode LBNO after final sintering at 1450 °C.

Value, at. %	O	Ni	Cu	Ba	La	Ce	Gd
Average	63	0	0	19	1.2	16	0.7
Standard deviation	4	0	0	2	0.3	1.5	0.1
Nominal BCGCuO composition	60	0	0.2	20	0	17.8	2

## Supplementary Materials

The following are available online at https://www.mdpi.com/1996-1944/12/16/2545/s1, Figure S1: X-ray diffraction (XRD) patterns of the micro-sized BaCeO_3_ (BCO) and BaCe_0.89_Gd_0.1_Cu_0.01_O_3−δ_ (BCGCuO) powders, Figure S2: XRD patterns of the micro-sized La_2_NiO_4+δ_-based (LNO) and La_1.7_Ba_0.3_NiO_4+δ_ (LBNO) powders, Figure S3: Morphology of the micro-sized BCGCuO powder after final milling, Figure S4: Morphology of the micro-sized BCO powder after final milling, Figure S5: Morphology of the micro-sized LNO powder, obtained by the solid state reaction method after final milling, Figure S6: X-Ray energy-dispersive (EDX) spectra for the BCGCuO film, deposited on the LNO substrate and sintered at 1450 °C (surface), Figure S7: EDX spectra for the BCGCuO film, deposited on the LNO substrate and sintered at 1450 °C (cross section), Figure S8: The overall EDX spectrum for the BCGCuO/BCO film, deposited on the LNO substrate (surface), Figure S9: EDX spectra for the BCGCuO film, deposited on the LBNO substrate and sintered at 1450 °C (surface), Table S1: Chemical analysis of the BCGCuO powder after the synthesis, in at. %, Table S2: Averaged chemical composition the BCGCuO film, deposited on the LNO substrate and sintered at 1450 °C in at. % (surface), Table S3: Averaged chemical composition the BCGCuO film, deposited on the LNO substrate and sintered at 1450 °C in at. % (cross section), Table S4: Averaged chemical composition of the the BCGCuO/BCO film, deposited on the LNO substrate (surface) (at. %).
