# Supplementary material for: Comparative Study of Electrophoretic Deposition of Doped BaCeO3-Based Films on La2NiO4+δ and La1.7Ba0.3NiO4+δ Cathode Substrates
Source: Materials (Basel). 2019 Aug 9;12(16):2545. doi: 10.3390/ma12162545 (PMC6720290; doi:10.3390/ma12162545)
Supplement: Supplementary file 1 [file materials-12-02545-s001.pdf]

materials

# Comparative study of electrophoretic deposition of doped $BaCeO_3$-based films on $La_2NiO_{4+\delta}$ and $La_{1.7}Ba_{0.3}NiO_{4+\delta}$ cathode substrates

**Elena Kalinina[1,2,*], Elena Pikalova[2,3,*], Alexandr Kolchugin[2,3], Nadezhda Pikalova[2,3] and Andrey Farlenkov[2,3]**

1 Institute of Electrophysics UB RAS, Yekaterinburg 620016, Russia
2 Ural Federal University, Yekaterinburg 620002, Russia
3 Institute of High Temperature Electrochemistry UB RAS, Yekaterinburg 620137, Russia
* Correspondence: jelen456@yandex.ru, Tel.: +7343-267-87-82 (E.K.); e.pikalova@list.ru, Tel.: +7343-362-31-94 (E.P.)

# Supplementary Materials

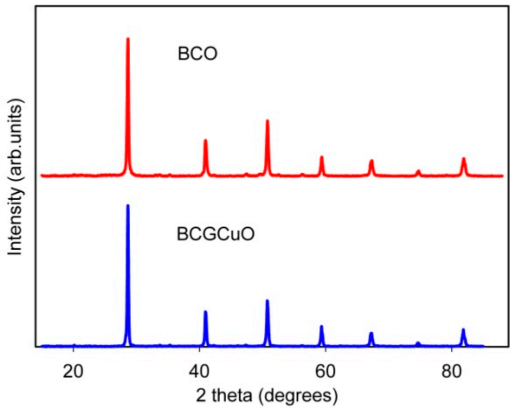


**Figure S1.** X-ray diffraction (XRD) patterns of the micro-sized $BaCeO_3$ (BCO) and $BaCe_{0.89}Gd_{0.1}Cu_{0.01}O_{3-\delta}$ (BCGCuO) powders.

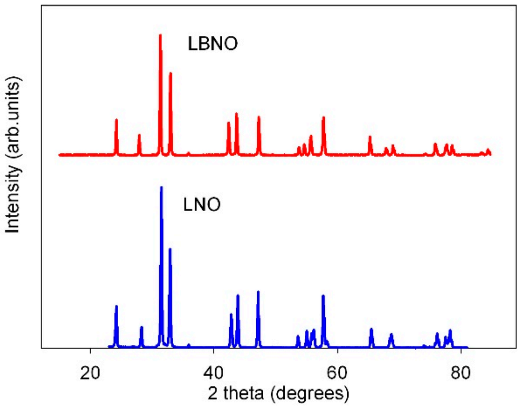


**Figure S2.** XRD patterns of the micro-sized $La_2NiO_{4+\delta}$-based (LNO) and $La_{1.7}Ba_{0.3}NiO_{4+\delta}$ (LBNO) powders.

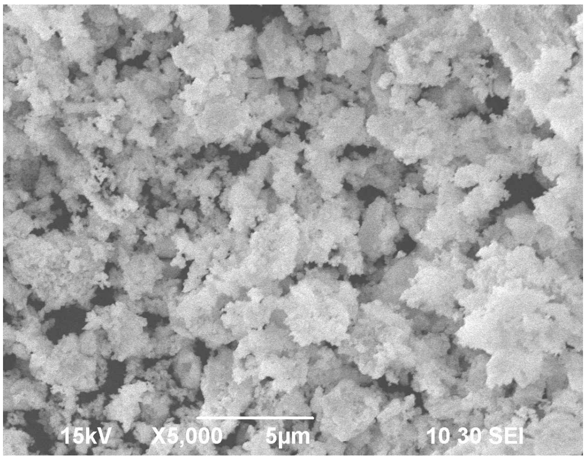


**Figure S3.** Morphology of the micro-sized BCGCuO powder after final milling.

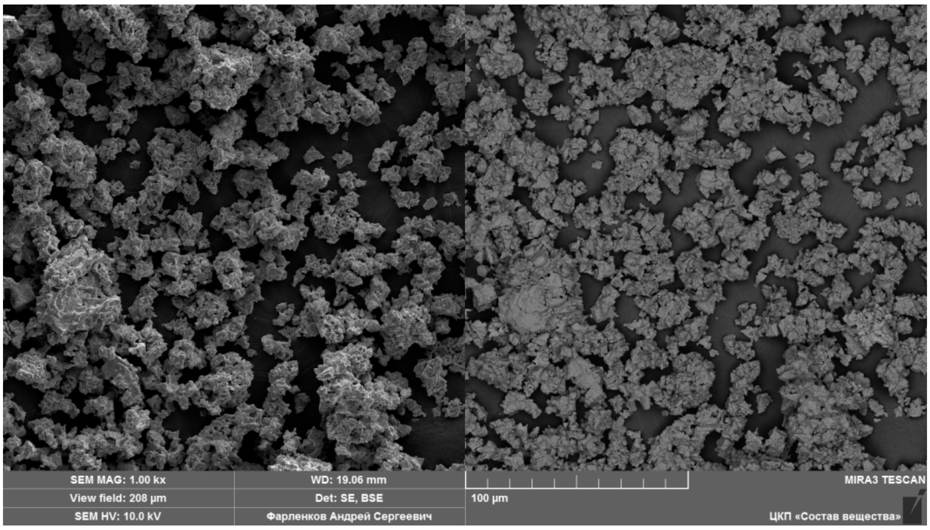


**Figure S4.** Morphology of the micro-sized BCO powder after final milling.

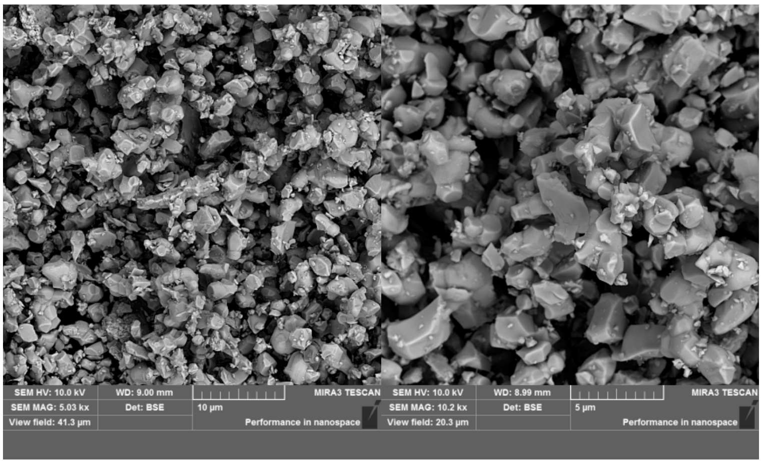


**Figure S5.** Morphology of the micro-sized LNO powder, obtained by the solid state reaction method after final milling.

**Table S1.** Chemical analysis of the BCGCuO powder after the synthesis, in at. %.

| Ba | 20.51 |
|---|---|
| Ce | 18.32 |
| Gd | 1.80 |
| Cu | 0.15 |

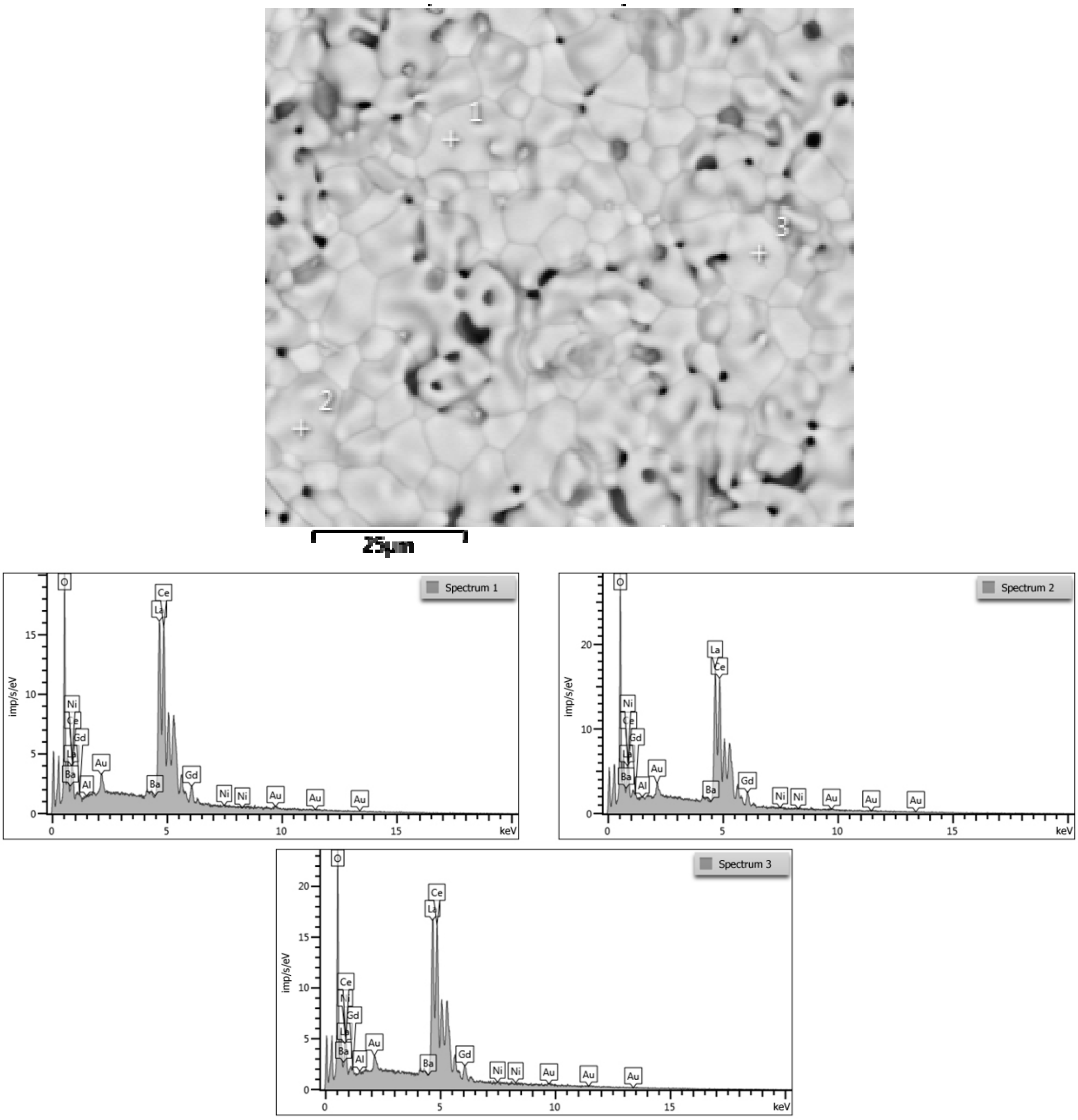


**Figure S6.** X-Ray energy-dispersive (EDX) spectra for the BCGCuO film, deposited on the LNO substrate and sintered at 1450°C (surface).

**Table S2.** Averaged chemical composition the BCGCuO film, deposited on the LNO substrate and sintered at 1450°C in at. % (surface):

| Elements | 1 spectrum | 2 spectrum | 3 spectrum |
|---|---|---|---|
| O | 61.24 | 66.76 | 63.76 |
| Al | 0.00 | 0.57 | 0.00 |
| Ni | 0.00 | 0.00 | 0.00 |
| Ba | 0.00 | 0.00 | 0.00 |
| La | 18.91 | 16.10 | 17.73 |
| Ce | 18.32 | 15.33 | 17.20 |

| | | | |
|---|---|---|---|
| Gd | 0.54 | 0.59 | 0.52 |
| Au | 0.99 | 0.66 | 0.79 |
| Sum | 100.00 | 100.00 | 100.00 |

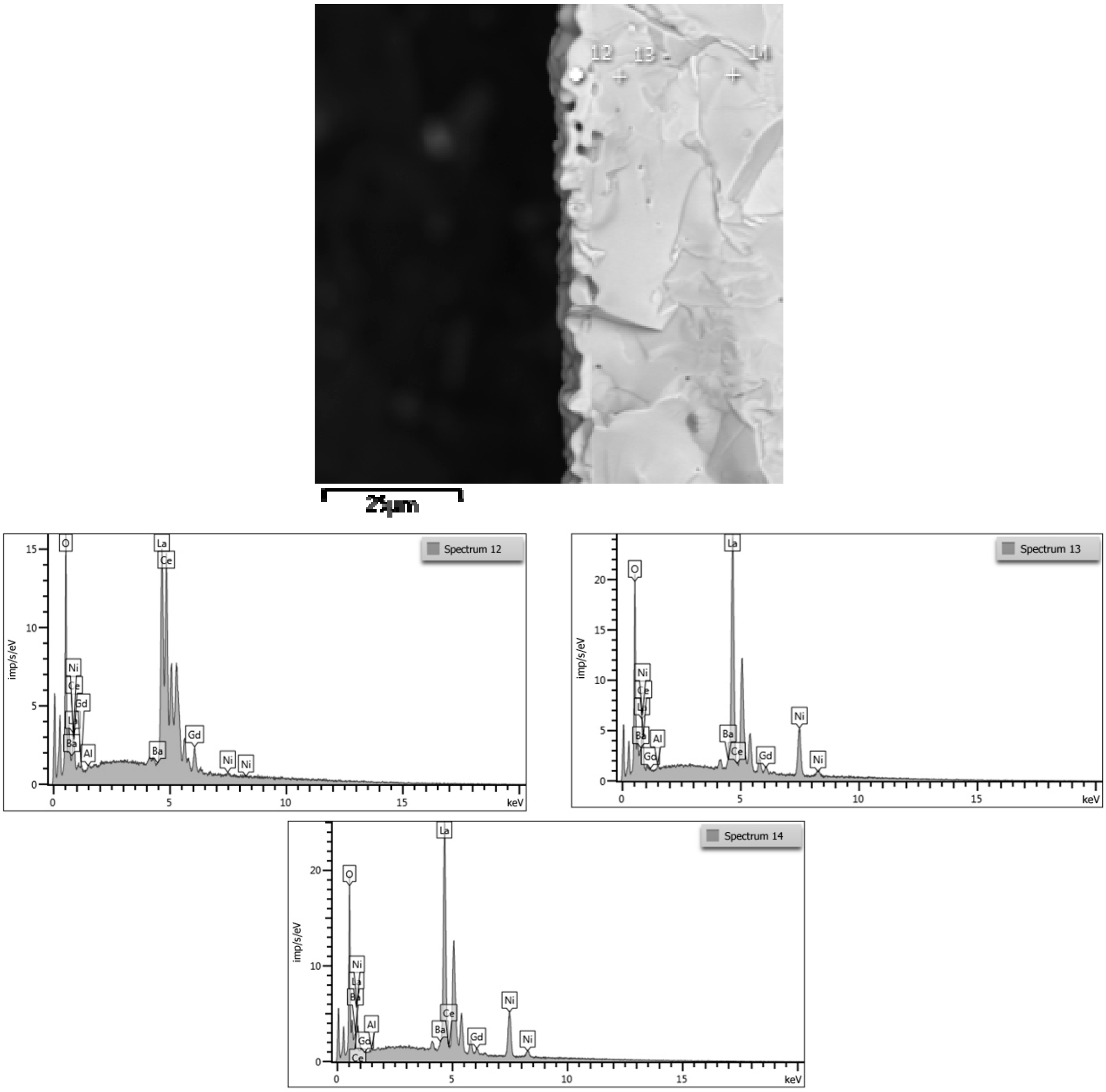


**Figure S7.** EDX spectra for the BCGCuO film, deposited on the LNO substrate and sintered at 1450°C (cross section).

**Table S3.** Averaged chemical composition the BCGCuO film, deposited on the LNO substrate and sintered at 1450°C in at. % (cross section):

| Elements | 1 spectrum | 2 spectrum | 3 spectrum |
|---|---|---|---|
| O | 57.14 | 56.60 | 54.98 |
| Al | 0.70 | 0.85 | 0.00 |
| Ni | 0.56 | 13.66 | 14.39 |
| Ba | 0.00 | 0.71 | 0.77 |
| La | 20.78 | 28.18 | 29.86 |
| Ce | 19.97 | 0.00 | 0.00 |
| Gd | 0.85 | 0.00 | 0.00 |
| Sum | 100.00 | 100.00 | 100.00 |

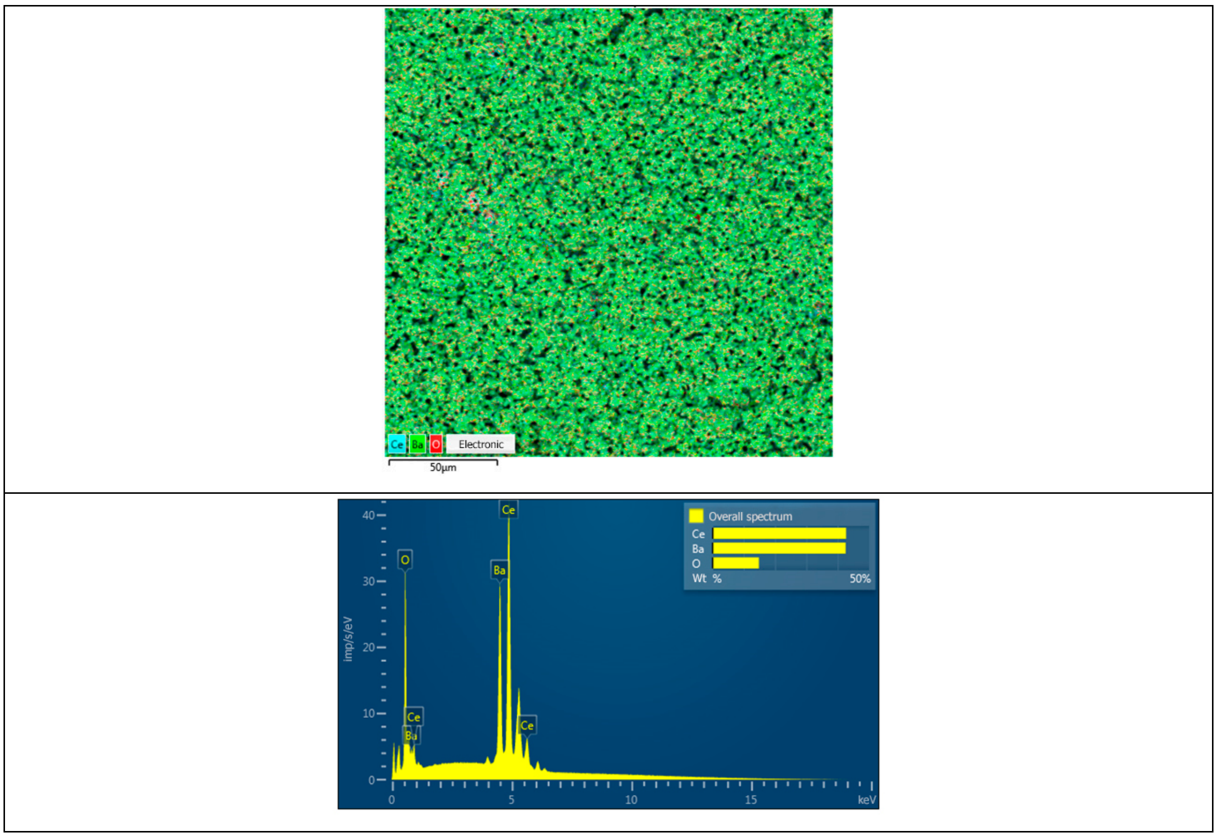


**Figure S8.** The overall EDX spectrum for the BCGCuO/BCO film, deposited on the LNO substrate (surface).

**Table S4.** Averaged chemical composition of the the BCGCuO/BCO film, deposited on the LNO substrate (surface) (at. %):

| Elements | Overall spectrum |
| --- | --- |
| O | 60.19 |
| Ba | 20.07 |
| Ce | 19.74 |
| Sum | 100.00 |

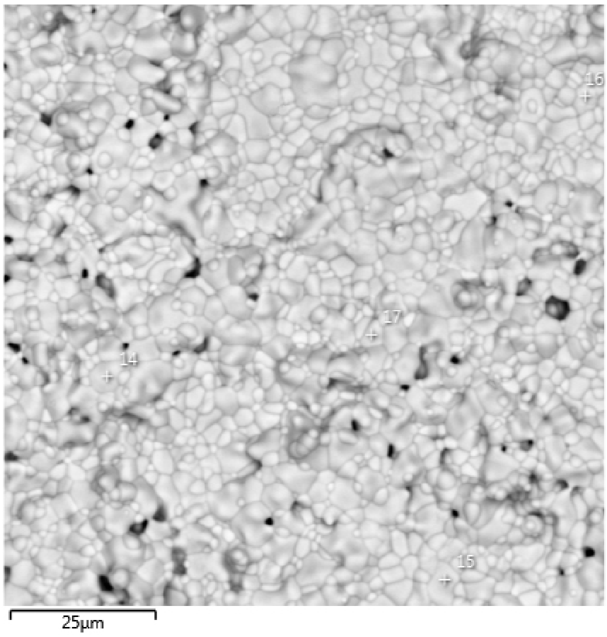

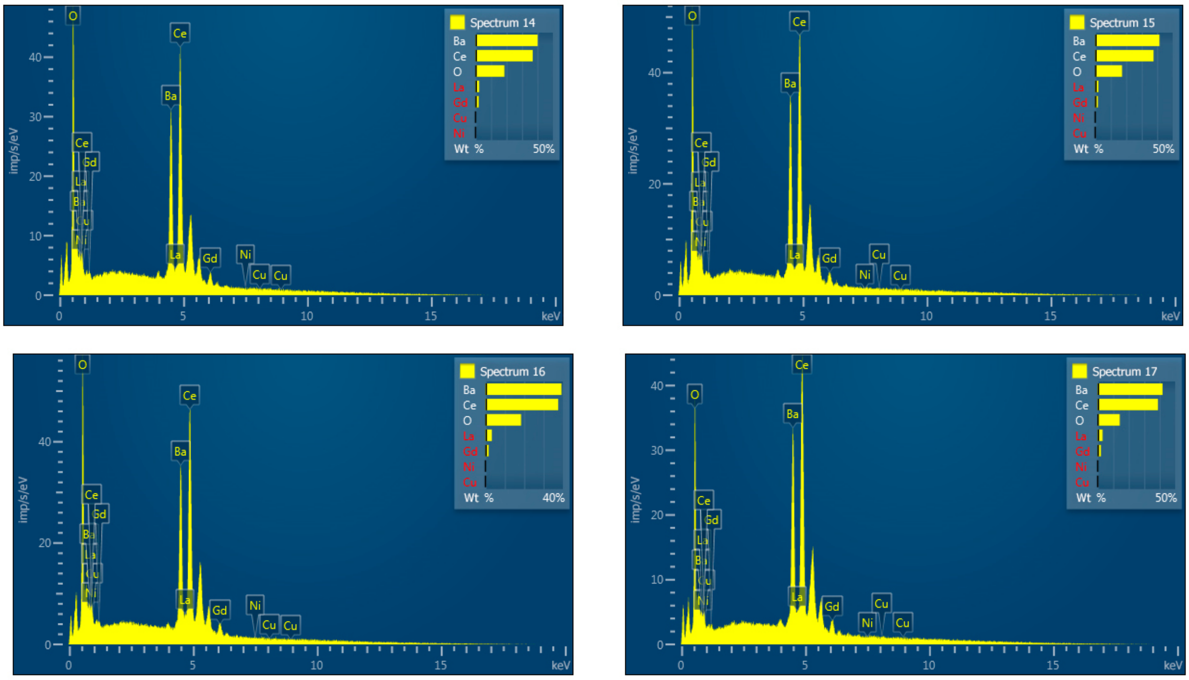


**Figure S9.** EDX spectra for the BCGCuO film, deposited on the LBNO substrate and sintered at 1450°C (surface).
